# Case Report: The effective treatment of patients in advanced no-small cell lung cancer patients with EGFR G719X/S768I/L861Q and acquired MET amplification: A case series and literature review

**DOI:** 10.3389/fonc.2023.1126325

**Published:** 2023-02-22

**Authors:** Yun Zhao, Cuiyun Su, Lina Shi, Wenqi Luo, Zhen Liu, Chuqiao Liang, Huilin Wang, Ruiling Ning, Qitao Yu, Wei Jiang

**Affiliations:** ^1^ Department of Medical Oncology of Respiratory, Guangxi Medical University Cancer Hospital, Nanning, Guangxi, China; ^2^ Medical Department, Nanjing Geneseeq Technology Inc., Nanjing, Jiangsu, China; ^3^ Department of Pathology, Guangxi Medical University Cancer Hospital, Nanning, Guangxi, China; ^4^ Department of Anesthesiology, Guangxi Medical University Cancer Hospital, Nanning, Guangxi, China

**Keywords:** uncommon mutation, EGFR G719X/S768I/L861Q, resistance, MET amplification, non-small cell lung cancer (NSCLC)

## Abstract

Preclinical cases suggest that EGFR tyrosine kinase inhibitors (TKIs) plus MET TKIs are a potential therapy for non-classical EGFR mutant lung cancers with MET amplification acquired resistance. Herein, we report for the first time the effectiveness of novel combination treatment regimens for patients with EGFR G719X/S768I/L861Q. Until the last follow-up assessment, two patients demonstrated improved survival after they switched to afatinib combined with savolitinib (PFS: 10 months) and furmonertinib combined with crizotinib (PFS: 6 months), respectively, that did not observed increased incidence and severity of adverse events. According to the findings of this study and literature review, various responses were observed from the combined therapy in NSCLC patients who harbored uncommon EGFR mutations and MET amplification. Furthermore, Next generation sequencing (NGS) leads to the discovery of uncommon of EGFR and reveals the co-mutations in NSCLC.

## 1 Introduction

Mutations of Epidermal growth factor receptor (EGFR) are one of the most prevalent oncogene drivers in non-small cell lung cancers. Up to 85 to 90% of EGFR-positive NSCLC patients carry the classical L858R and exon 19 deletions, and the remaining 10%-15% are defined as non-classical mutations (1). US Food and Drug Administration (FDA) approved therapies for non-classical EGFR mutations including G719X, S768I and L861Q, for which afatinib was deemed effective base on retrospective studies (2). MET amplification has been proven as a mechanism of acquired resistance in advanced NSCLC patients with EGFR G719X/S768I/L861Q (3, 4). A previous case report indicated that the combination of afatinib and crizotinib, the inhibitor of MET amplification, could be a promising strategy for reversing acquired resistance in patients with EGFR L861Q and MET amplification mutations (5), Nevertheless, limited evidence has demonstrated the efficacy of different combined therapy patterns in these patients. Therefore, we initiated a real-world study to investigate the distribution and therapeutic responses of NSCLC patients with EGFR G719X/S768I/L861Q and MET amplification who were treated under three different treatment patterns: afatinib plus crizotinib, afatinib plus savolitinib, furmonertinib plus crizotinib, which demonstrated significant antitumor efficacy and favorable clinical effect. In addition, there was a significant difference in progression free survival (PFS) across treatment regimens and EGFR alteration subtypes.

## 2 Case presentation

### 2.1 Case 1

**Figure 1:**
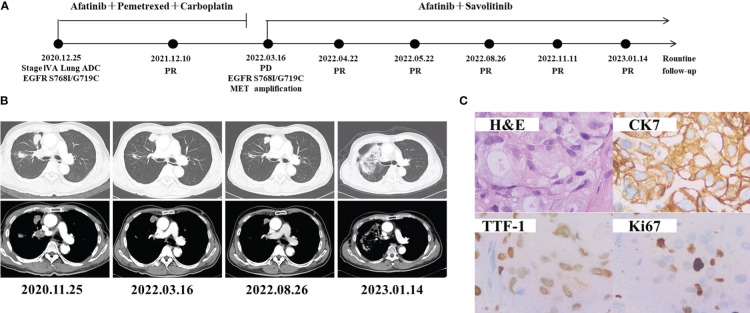
Schematic of treatment history of Case 1. **(A)** The timeline of treatment. **(B)** Chest CT images throughout the disease course. **(C)** Pathological examination of the surgical specimen. IHC testing (400×) results showed positive expression of TTF-1, CK7 and Ki67.

A 62-year-old man with 40 pack-year smoking history was admitted to our hospital due to right chest pain (**Figure 1A**). The computed tomography (CT) revealed a tumor in the right lung with multiple pulmonary metastases, right clavicle involvement and right neck lymph node metastasis on November 26, 2020 (**Figure 1B**). Bronchoscopic biopsy and Immunohistochemical (IHC) analysis revealed adenocarcinoma with positive expression of TTF-1, CK7 and Ki67 (**Figure 1C**). Examination of NGS (Geneseeq Technology Inc., Nanjing China) with a panel of 139 cancer-related genes by using a biopsy tumor tissue, and it was found *EGFR* G719C/S768I and *TP53* K320* mutations. He was diagnosed as adenocarcinoma in the upper lobe of the right lung (cT4N3M1a, stage IVA), and treated with Afatinib (30 mg once daily) combined with pemetrexed and carboplatin as the first-line treatment since Dec 25, 2020, and the best efficacy evaluation was partial response (PR). On Mar 16, 2022, chest CT showed enlarged pulmonary space-occupying lesion after 15 months of afatinib treatment. *EGFR* G719C/S768I, *MET* amplification (copy number 15), and *CDK4* amplification (copy number 20) were found by comprehensive genomic profiling of the CT-guided lung puncture specimen (**Table 1**). *MET* amplification and *CDK4* amplification were considered as the reasons of afatinib resistance. Given that the inhibitor of CDK4 has not been approved for lung cancer treatment, savolitinib (400 mg once daily) as the MET inhibitor were administered to the patient with the combination of afatinib (30 mg once daily). Chest CT scan 1 month later demonstrated obvious shrinkage (PR) and 2 months later it showed confirmed PR, Untill the last follow-up on 14 Jan 2023, the patient achieved 10 monthes PFS. Continuously decreasing levels of carcinoembryonic antigen (CEA) were observed. The patient recieved radiotherapy (48 Grey) during 2022.9.13-2022.10.6, due to the slow progression of leisions was observed in August. The last CT result revealed a Grade 1 radiation pneumonia. The treatment is still being continued, and the timeline of all treatment process for the patient is shown in **Figure 1**.

**Table 1:** Concurrent alterations identified in patients.

Case 1 (alteration, allele frequency)	Case 2 (alteration, allele frequency)	Case 3 (alteration, allele frequency)
EGFR:c.2155G>T (82.9%)	EGFR:c.2582T>A (84.9%)	EGFR:c.2156G>C (74.7%)
EGFR:c.2303G>T (82.78%)	MET amplification (CN:11.1)	MET amplification (CN:8.4)
MET amplification (CN:15)	EGFR amplification (CN:12.7)	EGFR amplification (CN:7)
CDK4 amplification (CN:20)	TP53:c.659A>G (45.9%)	TP53:c.218_219delTG (23.1%)
TP53:c.958A>T (79.08%)	AKT1:c.672C>G (5.8%)	BRIP1:c.2593C>T (11.4%)
ATR:c.3726-2A>G (21.38%)	CYP2D6:c.1010A> G (10.8%)	GRIN2A c.1318G>A (7.8%)
PTEN deletion (CN:1)		PIK3R1:c.374C>T (35.9%)
CCND2 amplification (CN:6)		RB1:c.261dupA (37.2%)
FAM135B amplification (CN:7)		
GLI1 amplification (CN:6)		
IL7R amplification (CN:6)		
MYC amplification (CN:8)		
PTK2 amplification (CN:8)		
EGFR amplification (CN:19)		

The copy number (CN) represents the specific amplification or deletion, and the normal genome is 2 copies.

### 2.2 Case 2

**Figure 2:**
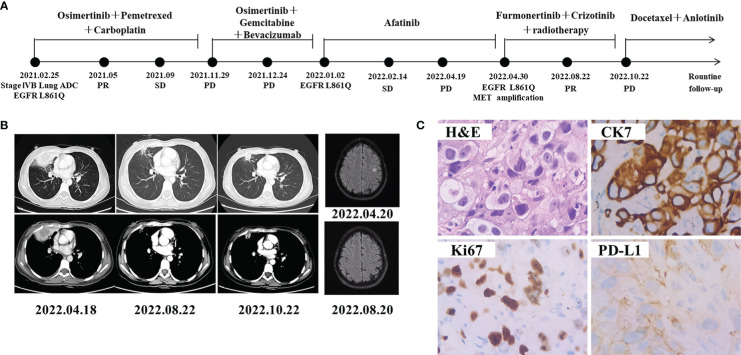
Schematic of treatment history of Case 2. **(A)** The timeline of treatment. **(B)** Chest CT images and brain MRI throughout the disease course. **(C)** Pathological examination of the surgical specimen. IHC testing (400×) results showed positive expression of CK7, PD-L1 and Ki67.

A 45-year-old Chinese female non-smoker was presented to our hospital with no family history of cancer suffered from repeated coughing on Feb 25, 2021 (**Figure 2A**). CT of the chest revealed a density mass in the right lung and multiple metastases in the mediastinal lymph nodes, vertebral lumbalis and right adrenal (**Figure 2B**). The level of the CEA was 16.27 ng/mL, which was much higher than the normal range of < 5.0 ng/mL. IHC analyses revealed that the tumor cells of the right lung were negative for TTF-1 and ALK, whereas focal staining was positive for CK7 and ki67 (**Figure 2C**). The patient was diagnosed with lung adenocarcinoma (cT3N3M1b, stage IVB) base on these findings. To identify a more effective treatment, NGS was performed on a right lung biopsy, and detected a rare *EGFR* L861Q co-occurring with *TP53* Y220C mutation. She was treated by osimertinib (80 mg, once a day) combined with pemetrexed and carboplatin as the first-line treatment since March 2021. However, the disease progressed after nine months as the follow-up CT scans showed enlarged lesions in middle lobe of right lung. The patient received osimertinib treatment with gemcitabine and bevacizumab for one month, but the disease progressed rapidly on December 27, 2021. The gene test result is the same as the last time, the patient was then switched to afatinib (30 mg, once a day) treatment starting from January 2022. Chest CT scan a month later demonstrated stable disease (SD) and 2 months later it showed PD. NGS profiling was performed using biopsy specimen of the lung. In addition to *EGFR* L861Q and *TP53* Y220C mutations, newly *MET* amplification (copy number 11.1) was detected (**Table 1**). Magnetic resonance imaging (MRI) scans revealed brain metastases (**Figure 2B**) on April 20, 2022. She switched to furmonertinib (80 mg, once a day) plus crizotinib (250 mg, twice a day) and whole-brain radiotherapy from April 28, 2022. PR was achieved after four months when CT scans showed shrinkages of target nodules in both lung and brain. The patient remained stable with a PFS for 6 months until PD on 22 October 2022, and then docetaxel plus anlotinib was started.

### 2.3 Case 3

**Figure 3:**
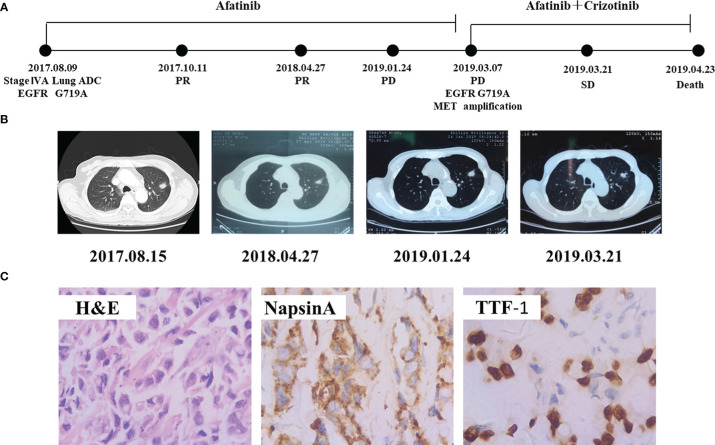
Schematic of treatment history of Case 3. **(A)** The timeline of treatment. **(B)** Chest CT images throughout the disease course. **(C)** Pathological examination of the surgical specimen. IHC testing (400×) results showed positive expression of Napsin A and TTF-1.

A 67-year-old Chinese male with no history of smoking visited our hospital in August 2017 with the chief complaint of dyspnea and pain in chest (**Figure 3A**). CT revealed a massive shadow in the upper lobe of the left lung, as well as metastases in both lungs and mediastinal lymph nodes (**Figure 3B**). Multiple bone metastases were also observed on a bone emission computed tomography (ECT) scan. CEA and cancer antigen 125 (CA-125) were detected at high levels of 100 ng/mL and 125.4 U/mL. The IHC analysis showed TTF-1(+), NapsinA (+), and ALK (−), Ki67 positive rate 40%, indicating lung adenocarcinoma (**Figure 3C**). The *EGFR* G719A mutation was detected by PCR. The patient was diagnosed with lung adenocarcinoma (cT1N2M1b, stage IVA) base on the findings above. He started to received afatinib (40 mg, once a day) since August 27, 2017. Chest CT scan 1month later demonstrated obvious shrinkage (PR) and 8 months later it showed confirmed PR (**Figure 3A**). The lung lesion was re-evaluated using CT scans on January 24, 2019, seven months following afatinib monotherapy, which revealed an increased tumor size. Comprehensive genomic profiling of the plasma identified *EGFR* G719A, *MET* amplification(copy number 8.4), and *TP53* V73Gfs*75 were found (**Table 1**). On March 7, 2019, he started to adopt the combination therapy of afatinib (40mg once a day) and crizotinib (250mg once a day). After 2 weeks treatment, CT revealed shrinkage of lesion in the left lung, and showed SD response. Unfortunately, the patient died due to no specific cause in April 2019.

## 3 Discussion

With the fast development of NGS and innovative medications, we may have started to avoid the exclusion of patients with uncommon or complex mutations who may benefit from anti-EGFR therapy. However, there are still some patients with non-classic EGFR mutation acquired resistance to EGFR-TKI. Some studies have suggested that MET amplification may be one of the main resistance mechanisms of afatinib in patients with EGFR G719X/S768I/L861Q (4). Abnormal activation of MET, which recruiting the MET receptor kinase to phosphorylate HER3 and activate the PI3K-Akt survival pathway, and thus, the PI3K-Akt pathway could be successfully blocked by inhibiting EGFR and MET simultaneously (6). The phase 1b TATTON study demonstrated that the combination of osimertinib and savolitinib had acceptable safety profile and promising antitumor activity in patients with MET amplification, classical EGFR mutations, advanced NSCLC, who had disease progression on a previous EGFR-TKI (7), illustrating that our understanding of single gene-targeted therapy has switched to associated genes and combined therapy. Nevertheless, evidence on the effectiveness of combined therapy among patients with non-classical EGFR mutations after acquiring MET amplification resistance are limited (**Table 2**).

**Table 2:** Reported combined therapy responses in lung cancer with EGFR G719X/S768I/L861Q and acquired MET amplification.

References	Sex	Age (years)	Clinical Stage	Concurrent EGFR Mutation	Treatment	Efficacy
4 (2)	male	41	cT1aN3M1	EGFR L861Q	Afatinib+Cirzotinib	Best response:PR; PFS:2.5 months
					Osimertinib+Crzotinb	Best response:SD; PES:7 months
5 (5)	female	54	cT4N3M1c	EGFR L861Q	Afatinib+Crizotinib	Best response:CR; PFS:10 months
Case 1	male	62	cT4N3M1a	EGFR G719C/S768I	Afatinib+Savolitinib	Best response:PR; PFS:10 months
Case 2	female	45	cT3N3M1b	EGFR L861Q	Furmonertinib+Crizotinib	Best response:PR; PFS:6 months

In our study, case 1 was treated with afatinib plus savolitinib to learn whether combined modality had a significant influence in the patient harboring *MET* amplification resistance with *EGFR* G719C/S768I mutations. Intriguingly, it could induce tumor remission with a PFS of more than 10 months. A case reported that an advanced NSCLC patient was diagnosed with acquired resistance of *MET* amplification (9.54 copy numbers) in the context of *EGFR* L861Q, and the combination therapy of afatinib plus crizotinib led to a 10 months PFS (5). A recent study also described a patient with concomitant *EGFR* L861Q and *MET* amplification at the time of initial diagnosis, who achieved PR after treating with afatinib and crizotinib with a PFS of 2.5 months, then he changed to osimertinib plus crizotinib due to brain metastases, and maintained SD for 6 months (4). Based on the computational modeling, furmonertinib might confer favorable activity to S768I and L861Q (8). Actually our study suggested that furmonertinib plus crizotinib showed potent efficacy in Case 2 with *EGFR* L861Q and *MET* amplification, she attained a 6 months PFS. In contrast, *MET* amplification in Case 3 with baseline *EGFR* G719A was found to be a driver of acquired resistance to afatinib. Treated by afatinib combined with crizotinib, the patient achieved disease control for only 1 months, yet he only experienced grade 1 heart rates drop. Patients harboring *EGFR* L861Q mutation maybe respond better than *EGFR* G719A to the combination therapy of afatinib and crizotinib. Last year, furmonertinib and savolitinib had granted approval in China for the NSCLC patients with classical EGFR mutation and MET exon14-skipping respectively (9, 10). These drugs are extensively utilized in China. Our cases provided additional treatment strategies for this subset of patients with EGFR G719X/L861Q/S768I and acquired MET amplification.

## 4 Conclusion

In line with our study, the acquired resistance mechanisms mediated by *MET* amplification, could be suppressed by unreported combination strategies involving targeted therapies, even when the concomitant non-classical *EGFR* mutation were G719C, S768I and L861Q. If our inference is validated by more cases, In this way, more favorable survival outcomes would be observed from combined therapy. Due to the heterogeneity and low prevalence of non-classical EGFR alterations, as well as the paucity of large-scale randomized clinical trials, clinical outcomes of diverse treatment modalities for NSCLC patient harboring non-classical EGFR alterations have not been fully elucidated. To guide precision therapy, further study is required to evaluate which treatment modality is the most effective for non-classical EGFR alterations. Of note, disease monitoring *via* NGS sequencing is crucial for targeted therapy in lung adenocarcinoma.

## Data availability statement

The original contributions presented in the study are included in the article/supplementary material. Further inquiries can be directed to the corresponding authors.

## Ethics statement

The studies involving human participants were reviewed and approved by the Ethical Committee of Guangxi Medical University Cancer Hospital. The patients/participants provided their written informed consent to participate in this study. Written informed consent was obtained from the individual(s) for the publication of any potentially identifiable images or data included in this article.

## Author contributions

YZ and CS were involved in the diagnostic flow and patient follow-up, LS wrote this article, and the manuscript was edited by CL. Collection and assembly of data: WL, ZL, HW, RN, QY and WJ. All authors contributed to the article and approved the submitted version.
